# Distinct satellite DNA composition between core and germline restricted chromosomes in *Bradysia* (*Sciara*) *coprophila*

**DOI:** 10.1093/g3journal/jkaf155

**Published:** 2025-07-03

**Authors:** Anne Kerrebrock, Jullien M Flynn, Robert B Baird, Christina N Hodson, Laura Ross, Yukiko M Yamashita

**Affiliations:** Whitehead Institute for Biomedical Research, Cambridge, MA 02142, United States; Whitehead Institute for Biomedical Research, Cambridge, MA 02142, United States; Whitehead Institute for Biomedical Research, Cambridge, MA 02142, United States; Howard Hughes Medical Institute, Cambridge, MA 02138, United States; UCL Department of Genetics, Evolution & Environment, University College London, London WC1E 6BT, United Kingdom; Institute of Ecology and Evolution, University of Edinburgh, Edinburgh EH9 3FL, United Kingdom; Whitehead Institute for Biomedical Research, Cambridge, MA 02142, United States; Howard Hughes Medical Institute, Cambridge, MA 02138, United States; Department of Biology, Massachusetts Institute of Technology, Cambridge, MA 02139, United States

**Keywords:** satellite DNA, germline-restricted chromosome, *Bradysia coprophila*, centromere

## Abstract

Programmed DNA elimination, a phenomenon wherein cells eliminate a subset of genetic material during certain stages of development, is observed in a broad range of organisms. The fungus gnat *Bradysia* (formerly *Sciara*) *coprophila* undergoes a series of programmed DNA elimination events during their development, including elimination of germline-restricted chromosomes (called L chromosomes) in the soma and elimination of paternal chromosomes during male meiosis. However, a lack of understanding surrounding the nature of eliminated chromosomes poses a barrier to studying programmed DNA elimination in this system. Highly repetitive satellite DNA, which often shows chromosome-specific distribution, is a possible candidate for sequences involved in programmed DNA elimination. In this study, we utilized recent genomic data and genome assemblies to identify new satellite DNA sequences of *B. coprophila*, and characterized their distribution on chromosomes. The results imply that the X and autosomes do not share centromeric satellite DNA sequence (BcopSat-155) with the L chromosomes. We further provide cytological evidence that confirms a recent finding based on the genome assembly that there are 2 distinct L chromosomes that were not previously distinguished cytologically. Together, our work lays a foundation for future studies to explore the possible connection between satellite DNA and the mechanism of programmed DNA elimination in *B. coprophila.*

## Introduction

A number of organisms eliminate a subset of their genetic material at specific stages of development, a process known as “programmed DNA elimination” or PDE. PDE may occur via chromosome elimination, wherein the entire chromosome(s) are lost, or chromosome diminution, wherein parts of chromosomes are deleted (reviewed in Zagoskin and Wang 2021; Dedukh and Krasikova 2022; Drotos *et al.* 2022). Some examples of PDE occur in the germline, where, for example, chromosomes of a particular parental origin are eliminated (reviewed in Herbette and Ross 2023). Other examples occur during embryogenesis and result in elimination of genetic material from the soma but retention in the germline, e.g. in hagfish, lamprey, nematode, and songbird (Tobler 1986; Nakai *et al.* 1991; Smith *et al.* 2009; Torgasheva *et al.* 2019). The biological significance of PDE remains largely a mystery: Why do cells eliminate DNA of a certain parental origin? What is the function of the eliminated DNA in the cells that retain it? It has previously been speculated that germline-limited genetic material may have important germline-specific functions (Bryant *et al.* 2016; Feng *et al.* 2017; Wang *et al.* 2017; Kinsella *et al.* 2019; Marlétaz *et al.* 2024) or that PDE is a mechanism to eliminate excess repetitive DNA from somatic cells, including satellite DNA (Kubota *et al.* 2001; Wang *et al.* 2017; Timoshevskiy *et al.* 2019) and transposable elements (Sun *et al.* 2014) to avoid deleterious effects on cell growth or development. PDE is also involved in sex determination and dosage compensation in certain organisms (Streit *et al.* 2016; Hodson and Ross 2021; Smith *et al.* 2021). Understanding the function or significance of PDE and/or DNA that is eliminated via PDE requires the knowledge of the mechanisms that underpin this phenomenon.

**Fig. 1.**
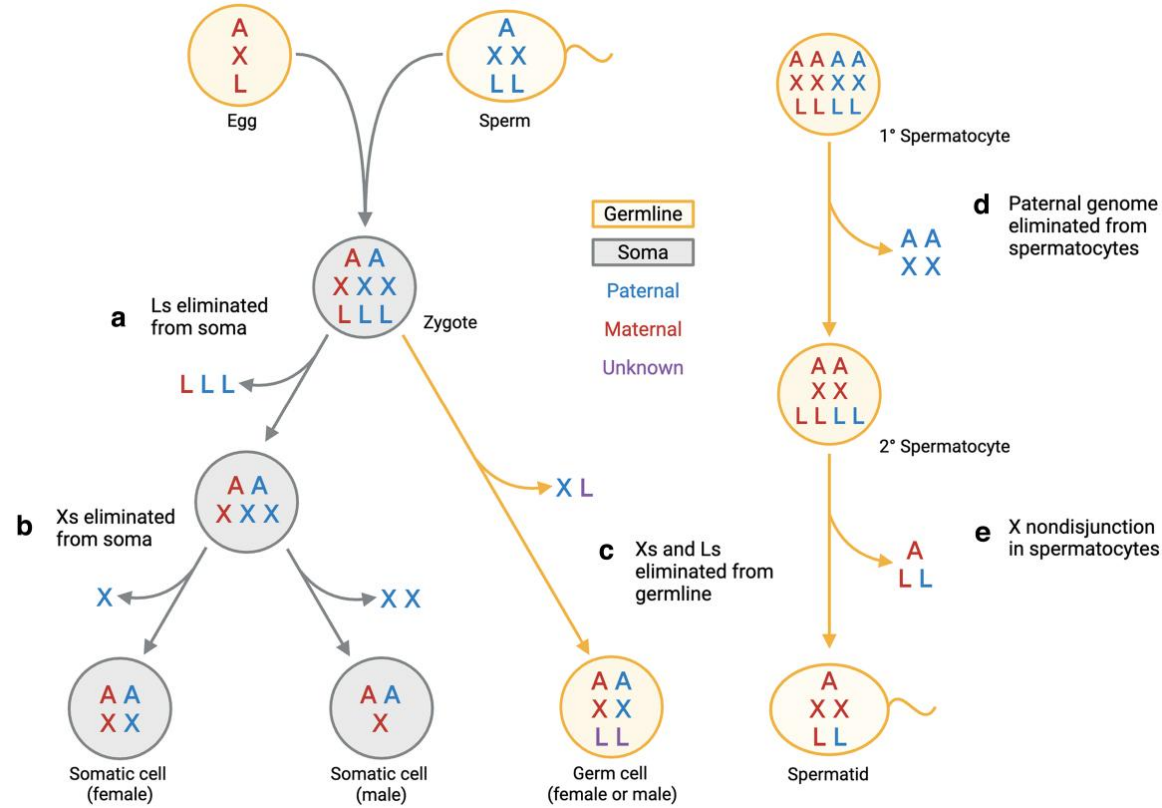
Chromosome eliminations in *Bradysia coprophila*. a–c) Chromosome eliminations in the early embryo. Upon fertilization, all embryos in this species start with 3 X chromosomes, 3 L chromosomes, and 2 sets of autosomes (XXX LLL AA), where sperm contribute 2 Xs, 2 Ls, and 1 set of autosomes (XX LL A), whereas eggs contribute a haploid genome (X L A). In early embryogenesis, germline-restricted L chromosomes are eliminated from somatic cells a) shortly followed by X chromosomes b). The embryo develops as a female or male if 1 or 2 X chromosomes are eliminated, respectively. One X and 1 L are eliminated in the germ cells of both sexes during late embryogenesis c). In male meiosis, the paternally-inherited X chromosome and autosomes are eliminated in the first meiotic division d). In the second meiotic division, the X undergoes nondisjunction, resulting in 2 X chromosomes in the single spermatid e). Note that homologous chromosomes (paternal vs maternal) are indicated by different colors. The same color indicates sister chromatids.

The fungus gnat *Bradysia* (formerly *Sciara*) *coprophila* has long served as a model system to study PDE because they undergo multiple types of chromosome elimination: elimination of germline-restricted chromosomes (GRCs, called L chromosomes in *Bradysia*) from somatic cells during embryogenesis and elimination of paternal chromosomes from male germ cells during meiosis (Fig. 1) (reviewed in Gerbi 2022; Du Bois 1933; Metz 1938; Rieffel and Crouse 1966; de Saint Phalle and Sullivan 1996; Perondini and Ribeiro 1997). Although PDE has been known to occur in *Bradysia* for many decades, the precise cellular and molecular mechanisms remain unknown, and a lack of understanding of the nature of the L chromosomes and how they are distinct from the other chromosomes prohibits further investigation into how they are eliminated.

Satellite DNA sequences are highly repetitive tandem arrays of noncoding DNA which often constitute a substantial proportion of the genome and is a major component of heterochromatin (reviewed in Garrido-Ramos 2017; Šatović-Vukšić and Plohl 2023). Some satellite DNA sequences are specific to individual chromosomes and/or species, therefore often serving as useful markers for chromosomes or species identification (Lohe *et al.* 1993; Lohe and Roberts 2000; Jagannathan *et al.* 2017). However, their highly repetitive nature often imposes a challenge in genome assembly, and the location and extent of satellite DNA within the genome are not always clear. In *B. coprophila*, 2 satellite DNAs were identified by isopycnic centrifugation that localize to centromeres, but their sequences remain unknown (Abbott *et al.* 1981). Subsequently, 3 other satellite DNA families were isolated, sequenced, and mapped to their chromosomal sites (Escribá *et al.* 2011). The 3 satellite DNA families were among the repeated sequences described in the recent genomic analyses of *B. coprophila* (Urban *et al.* 2021, 2025) Highly repetitive DNA has been shown to be a target of somatic chromosome elimination in several species including hagfishes, lampreys, and parasitic nematodes (Kubota *et al.* 2001; Wang *et al.* 2017; Timoshevskiy *et al.* 2019; Zagoskin and Wang 2021), thus characterization of satellite DNA in *B. coprophila* may also provide insights into mechanisms of PDE in this species.

In this study, by analyzing recent genomic data of *B. coprophila*, we identified several new satellite DNA families, thus broadening the repertoire of known satellite DNA sequences in this species. We found that some satellite DNAs were clustered near the centromeres and ends of chromosomes, while others were broadly interspersed across the genome. Our analysis revealed 2 interesting features of *B. coprophila* chromosomes. First, we show that a 155 bp satellite DNA, which is found on the centromeres of the core chromosomes (X and autosomes) (Escribá *et al.* 2011), is absent from the germline-restricted L chromosomes. Second, we found that the 2 L chromosomes, which were previously not distinguished cytologically, are 2 distinct chromosomes with distinct satellite DNA compositions. This confirms a recent study that suggested, based on genomic data, that the 2 L chromosomes are distinct from each other (Hodson *et al.* 2022). Our findings also show that satellite DNA composition is distinct on the germline-limited L chromosomes relative to the core chromosomes, and prompt further study of the potential relationship between satellite DNA and chromosome eliminations in *B. coprophila*.

## Materials and methods

### Fly culture stocks and maintenance

The *B. coprophila* HoLo2 and 6980 stocks were obtained from Dr. Susan Gerbi at Brown University. Flies were maintained as described in Gerbi (2024). There are 2 distinct X chromosomes in *B. coprophila* females, X′ and X, which differ by the presence of multiple paracentric inversions (Crouse 1979; refer to Baird *et al.* (2023b) for a comparison of the assemblies of the X′ and X chromosomes). Sex determination in *B. coprophila* is maternally determined: X′X females produce only daughters (either X′X or XX), and XX females produce only males. Males do not carry the X′ chromosome, and are XO in the soma and XX in the germline. Eliminations of the L chromosomes and the extra paternal X chromosome occur identically in X′X and XX females. Using DNA fluorescence in situ hybridization (FISH) on polytene chromosomes, we found that the X′ chromosome mostly carried the same satellite DNA complement as the X chromosome. However, it was difficult to determine the location of the satellite DNA arrays in X′X females due to the presence of the inversions. For this reason, all images shown contain polytene chromosomes from either XX females or XO males.

### Satellite DNA identification

We used 2 methods to identify satellite sequences. First, we used Tandem Repeats Finder (TRF; Benson 1999), to identify satellites in the assembly of the core chromosomes (Urban *et al.* 2021) and the genome assembly that contained L chromosomes (Hodson *et al.* 2022) using parameters optimized for satellite repeat units of 20–500 bp (Supplementary Fig. 1). Second, we searched for simple short (≤20 bp) satellites in raw Illumina WGS reads using k-seek (Wei *et al.* 2014), but using this method, we did not identify any such satellites enriched in germline short reads, and could not identify sequences with over 10 kb total abundance in somatic reads. We filtered repeat calls to only those in tandem arrays of at least 5 copies, and we standardized the rotation/reverse complement of the consensus sequence of each array. We then used cdhit (Li and Godzik 2006) to merge related sequences into 190 clusters representing different satellite families. We selected the 8 most abundant satellite families (present at ≥200 kb in the assembly) for further analysis. We generated a BED file of all contigs containing tandem repeats from the raw TRF output to analyze the locations and abundances of the 8 repeats selected. Satellite families were named using a modification of the naming convention recommended by de Lima and Ruiz-Ruano (2022): (species abbreviation)Sat—(unit length), i.e. “BcopSat-145” indicating a satellite DNA family in *B. coprophila* with a 145 bp unit repeat. We did not use abundance ranking, as we were not able to determine the abundance for some of the satellite DNA families that we identified, especially the satellites enriched on the L chromosomes. Additionally, in *Drosophila*, satellite DNA family abundance has been shown to vary among strains (Wei *et al.* 2014), cautioning us from naming satellite DNA based on its abundance.

### DNA FISH

DNA FISH was performed on chromosome squashes, using a protocol modified from Larracuente and Ferree (2015). Sample preparation: For salivary gland polytene chromosome preparation, salivary glands were dissected from fourth instar larvae in 45% acetic acid and moved to 12 μl of 45% acetic acid on a clean Superfrost slide (VWR). A coverslip was added, and the sample was gently tapped to spread the chromosomes. A rubber stamp was then used to manually squash the preparation, and the sample was frozen by submerging it in liquid nitrogen. Subsequently, the coverslip was removed and the slides were washed in 100% ethanol and air-dried. Slides were fixed in 3.7% formaldehyde in PBS for 4 min at room temperature. The slides were washed in PBS, 2× SSC, and dehydrated in 70% ethanol and 100% ethanol. Slides were store in a dark, dust-free location until hybridization. For meiotic chromosome spreads, testes were dissected from young pupae (1/3–1/2 filled eyespots) in PBS. The testes were moved to 20 μl of 2.2% formaldehyde in 45% acetic acid, and fixed for 4 min at room temperature. A coverslip was placed onto the sample, and the sample was squashed by applying the rubber stamp onto it. The sample was then frozen in liquid nitrogen, and the coverslip was removed. The slide was rinsed in 100% ethanol, and air-dried. Prior to hybridization, the sample was treated with RNase A (2 mg/ml) for 10 min at 37 °C, and subsequently washed in PBS, and dehydrated in 70% ethanol then 100% ethanol, and air-dried. Slides were stored as above. Hybridization: Oligos (30–40 mer; Integrated DNA Technologies) labeled with Alexa-488, Cy3, or Cy5 at the 5′ end were used as probes (probe sequences are provided in Table 1). A total of 20 μl of hybridization buffer (50% formamide, 10% dextran sulfate, 2× SSC, and 0.5 mM of each probe) was added onto the dry sample, and a coverslip was placed onto the sample. The sample was treated either at 91 °C for 3 min (salivary glands) or at 95 °C for 5 min (testes squashes) using a digital dry bath (USA Scientific) to denature DNA. The slides were cooled briefly, covered with parafilm and placed in a humid chamber in the dark, and incubated overnight at room temperature. The next day, the coverslips were removed, and the slides were washed at room temperature in 2× SSC (2 times for 10 min) followed by 0.2× SSC (once for 10 min) for salivary glands, or in 0.1× SSC (3 times for 15 min) for testes squashes. The slides were air-dried in the dark, and mounted using Vectashield Antifade Mounting Medium with DAPI (Vector Laboratories). Images were taken using a Leica SP8 confocal microscope with a 63× oil immersion objective and processed using ImageJ software (Fiji).

**Table 1.** Abundant satellite families in *Bradysia coprophila*.

Satellite family	Monomer length (bp)	Array size (kb)	Amount in assembly (kb)	Chromosomal location by DNA FISH	Distribution pattern	G-C content	Probe sequences
BcopSat-145	145	0.7–12	1,923 (0.62%)	X, II, III, IV, L	Chromosome ends plus weaker dispersed bands	35.86% 38.89%	145A GGGTCCTCGGACTAAGGCCGCTACTAGGGCCCTATGTGT 145B GGGCCCTTGGACTAAGGCCGCTACTCGGCCCTAAGTGT
BcopSat-155/Sccr	155	0.6–43	1,500 (0.48%)	X, II, III, IV (not L)	Centromeric	27.74%	155 ACTGCGCACCGTTGAAACACACTAAAAACTCACTTGCCT
BcopSat-176	176	0.9–36	416 (0.13%)	X, II, IV (not L)	Dispersed bands	27.84% 28.00%	176A AGCATAGGCTTAGCATACATTTTCTTAATAATT 176B CAAGCATAGGCGTAGCAAACATTTTCTTAATA
BcopSat-37	37	0.6–29	408 (0.13%)	X, II, III, IV (not L)	Dispersed bands	24.32%	37A AAAATAATTAATTTAAACGTCGAAATTCGTCATTTGG 37B AAAATAATTAATTTAAACGTCGAATTTCATCGTTTGG
BcopSat-109	109	0.5–28	392 (0.13%)	X, II, III, IV (weak L)	Chromosome ends plus weaker dispersed bands	41.28%	109 GGAAGTTAGGAAGTGCCAGAGGAATCATCTGTCAT
BcopSat-162	162	0.6–41	388 (0.13%)	X, II, III, IV	Centromeric, pericentromeric, and chromosome ends	24.69%	162 GCCATACATTATGACCAAAAAATGGCATTGTT
BcopSat-129	129	0.6–37	375 (0.12%)	X, II, III (weak), IV, L	Dispersed bands	42.64%	129 GTCACTCACACACAAAAGCGTACACTGTGGTCAC
BcopSat-94	94	0.6–10	197 (0.06%)	X, II (weak), III, IV (not L)	Pericentric on III and IV Dispersed bands	41.49%	94 TCGGTCTCTTACATTCTCGTAAAATATTCAAAA

## Results

### Identification of new satellite DNA families in the *B. coprophila* genome

Previously, 3 *B. coprophila* satellite DNA repeats were identified by microdissection of the pericentromeric region of the X chromosome, followed by cloning of the microdissected DNA into a plasmid vector (Escribá *et al.* 2011). This approach identified a 42 bp satellite DNA confined to the proximal heterochromatin on the X chromosome, a 382 bp satellite DNA present on the X, IV, and L chromosomes, and a 155 bp satellite DNA (Sccr) at the centromeres of all chromosomes (Escribá *et al.* 2011). Because these 3 satellite DNAs likely only represent a small fraction of satellite DNAs of *B. coprophila*, we utilized recent genome sequence data to identify more satellite DNAs.

Using the *B. coprophila* core chromosome (i.e. X and autosome) scaffolds (Urban *et al.* 2021) and applying the Tandem Repeats Finder software (Benson 1999), we found that tandem repeats make up approximately 5.5% of the genome assembly. We found 651 satellite DNA repeat sequences which had a unit size between 30 and 180 bp (workflow summarized in Supplementary Fig. 1). These 651 satellite DNA sequences were clustered into 190 satellite DNA families based on the sequence homology. We selected the 8 most abundant families (each family spanning 200 kb to almost 2 Mb in the assembly) to further analyze their distribution within the genome. The total amount of the 8 most abundant satellite DNAs summed to 5.6 Mb (Table 1, representative sequences of the repeat units are in Supplementary Table 1). These 8 satellite DNA families were named using species abbreviation/Sat (BcopSat) followed by the repeat unit length (Table 1) (see Materials and methods for details). Among these was the 155 bp “Sccr repeat” family described previously (Escribá *et al.* 2011), here referred to as BcopSat-155. The other 7 satellite DNA families are newly described, and have unit repeat lengths between 37 and 176 bp (Table 1). Each satellite DNA family contains various minor sequence and length variants, although the repeat divergence landscape shows a high degree of homology (>90% sequence identity) within families (Supplementary Fig. 2). The satellite DNA families BcopSat-145, BcopSat-176, and BcopSat-37 had 2 abundant sequence variants, which we will refer to as A and B (e.g. BcopSat-145A and BcopSat-145B). Two of the previously identified satellite DNAs, with 42 and 382 bp repeat units, had low abundance in the assembly (8 and 42 kb total, respectively) (Escribá *et al.* 2011). Together, these analyses identified highly abundant satellite DNAs in the *B. coprophila* genome, totaling 5.6 Mb (1.7% of the assembly), most of which are identified for the first time (Table 1).

### Cytological mapping of satellite DNA on the *B. coprophila* polytene chromosomes

To characterize the chromosomal distribution of the newly identified satellite DNA families, we conducted DNA FISH on polytene chromosomes from the salivary glands. *B. coprophila* salivary polytene chromosomes are amplified up to 8192 times by endoduplication (Rasch 1970), allowing the detection of even low abundance DNA sequences that may be undetectable in diploid cells (Zhimulev and Koryakov 2009). Oligo probes for DNA FISH were designed using conserved DNA sequences within the family, and were thus expected to detect most (if not all) of the satellite DNA variants within each family. For satellite DNAs that had 2 major variants, namely BcopSat-145, BcopSat-176, and BcopSat-37, we designed probes that are expected to be specific to each variant (Table 1). Indeed, these variant-specific probes were able to discriminate between the 2 major variants when both probes were used together, although each probe hybridized to both variants when used alone in the absence of competing probes with a higher specificity (Supplementary Fig. 3).

**Fig. 2.**
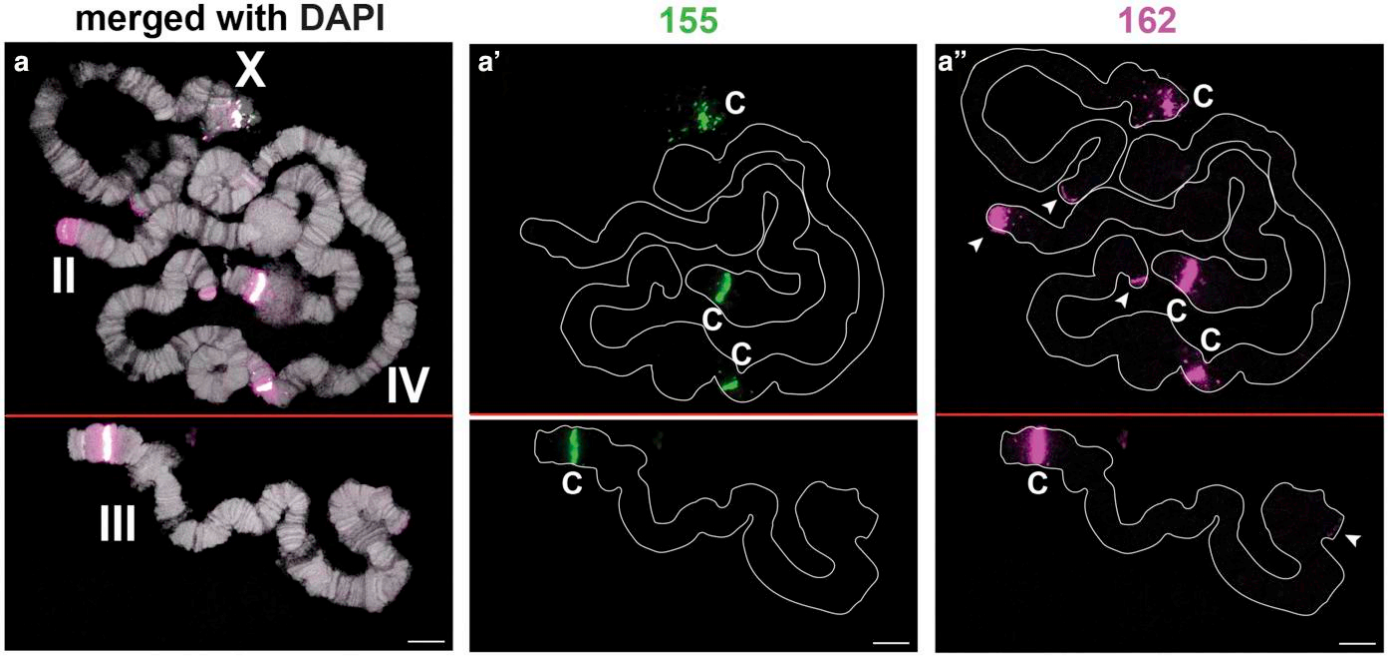
Centromeric and pericentromeric satellite DNA distributions. Distribution of BcopSat-155 (green) and BcoSat-162 (magenta) families is shown (a′ and a″ without DAPI). Both probes hybridize to centromeric and pericentromeric regions, respectively. BcoSat-162 also hybridizes to telomeric regions of all 4 chromosomes (arrowheads). Putative centromeres are indicated by “c”. Scale bar represents 10 µm.

**Fig. 3.**
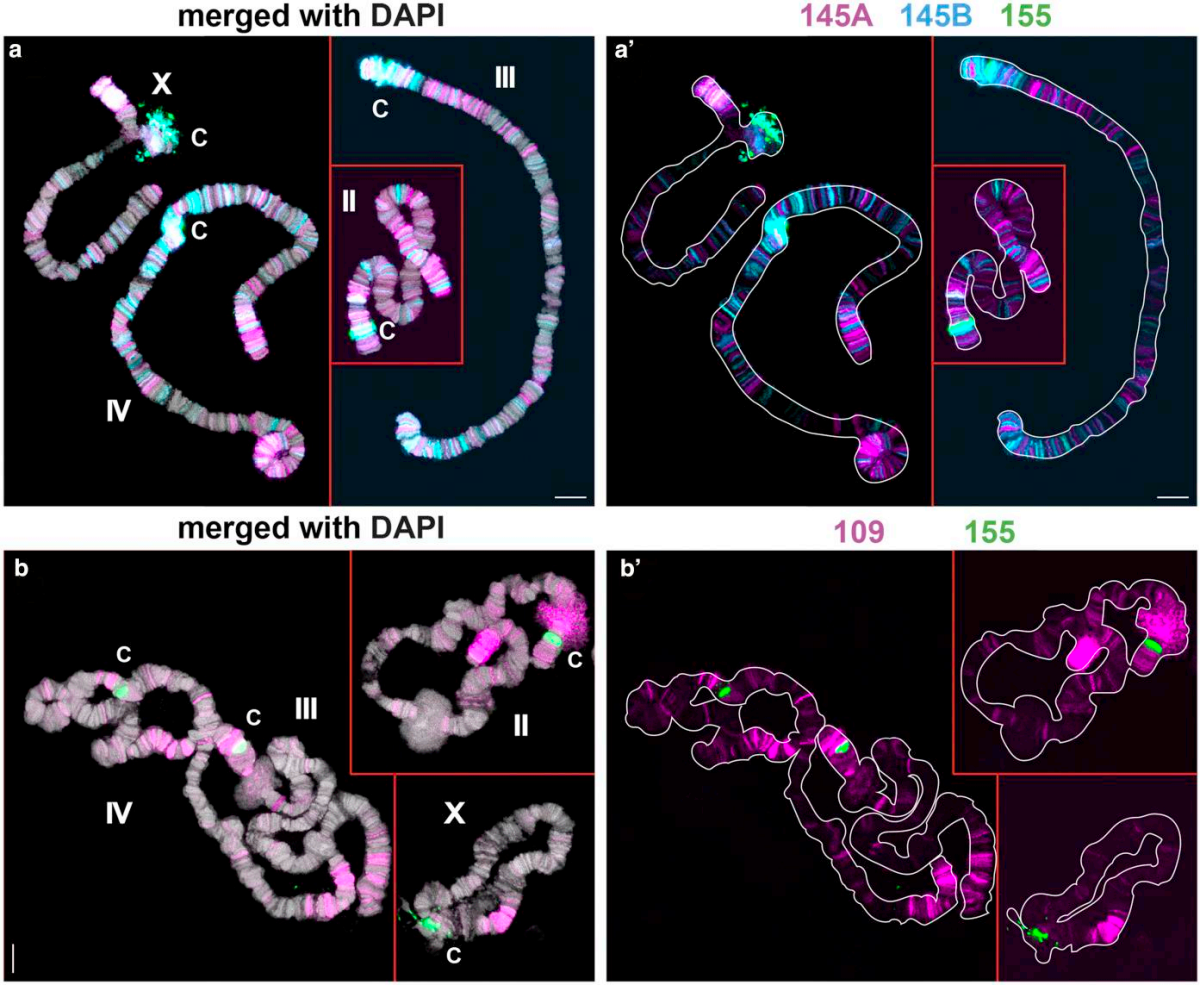
Ubiquitous distributions of satellite DNAs. a) Hybridization of BcopSat-145. The 145A (magenta) and 145B (blue) variant sequences are most abundant at the chromosome ends and also weakly at the pericentromeric region of chromosome IV (a′ without DAPI). Numerous light bands are present along the arms of all 4 chromosomes. b) Hybridization of BcopSat-109, which is strongest at or near the chromosome ends, with many lighter bands along the chromosome arms (b′ without DAPI). This satellite is also present within DNA puff II/2B adjacent to the centromeric region. Centromeric regions are shown by BcopSat-155 (green, c). Scale bar represents 10 µm.

**Fig. 4.**
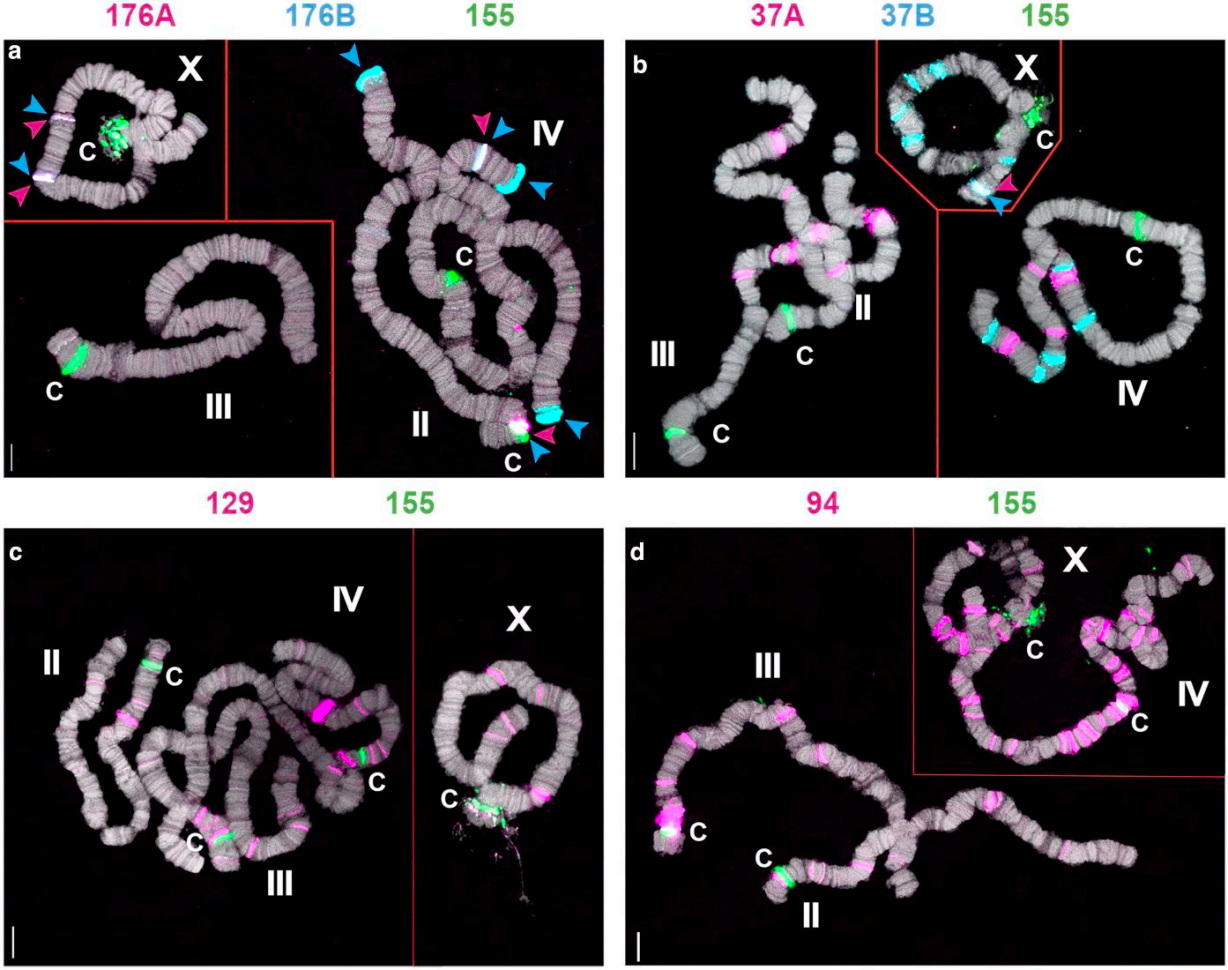
Scattered band distribution of satellite DNA. a) Hybridization of the BcopSat-176 family. The 176A (magenta) and 176B (blue) probes overlap at all internal bands, but only 176B is found at the chromosome ends. b) Hybridization of the BcopSat-37 family. The 37A (magenta) and 37B (blue) probes show that these 2 variants do not overlap, except for 1 band on the distal end of the X (blue and magenta arrowheads). c) Hybridization of the BcopSat-129 family. The 129 probe (magenta) is present in light bands on the arms of all 4 chromosomes, with 1 strong band on chromosome IV. d) Hybridization of the BcopSat-94 family. The 94 probe (magenta) is abundant in the centromeric and pericentromeric regions of chromosomes III and IV, and in additional lighter bands on the arms of all 4 chromosomes. Centromeric regions are shown by BcopSat-155 (green, c). Scale bar represents 10 µm.

DNA FISH to polytene chromosomes confirmed the presence of these satellite DNAs and revealed their distribution along the chromosomes (Figs. 2–4, results summarized in Table 1). We confirmed that BcopSat-155/Sccr is indeed invariably localized to the centromeres of core chromosomes (Escribá *et al.* 2011) (Fig. 2). We found that a second satellite family, BcopSat-162, colocalizes with BcopSat-155 at the centromeres and extended into the pericentromeric region beyond the BcopSat-155/Sccr signal (Fig. 2). BcopSat-162 exhibited additional FISH signals on the distal end of II, and in lower abundance on the other chromosome ends (Fig. 2a″, arrowheads).

Other satellite DNAs were distributed outside the centromeres. BcopSat-145, the most abundant satellite DNA family in the genome (Table 1), was observed to be dispersed in many shorter arrays along the chromosome arms (Fig. 3, a and a′). The 2 variants of BcopSat-145 (BcopSat-145A and BcopSat-145B) were detected at both overlapping and distinct locations along the chromosomes.

BcopSat5-109 was mostly concentrated at or near the chromosome ends with many additional weaker bands along the length of the chromosomes (Fig. 3, b and b′). BcopSat-176, BcopSat-37, BcopSat-129, and BcopSat-94 hybridized to fewer discrete bands, often along the chromosome arms (Fig. 4). BcopSat-176A and BcopSat-176B variant satellite DNAs were found in some nonoverlapping bands, suggesting that each variant occupies distinct locations within the genome (Fig. 4, a and a′). The 2 variants of BcopSat-37 (BcopSat-37A and BcopSat-37B) rarely overlapped (Fig. 4, b and b′). BcopSat-129 families had scattered bands along the arms of the 4 chromosomes (Fig. 4, c and c′). The BcopSat-94 was abundant in blocks in the pericentromeric regions of chromosomes III and IV and in many scattered bands along the chromosome arms (Fig. 4, d and d′).

### Core chromosomes and L chromosomes have distinct centromeric satellite DNA

**Fig. 5.**
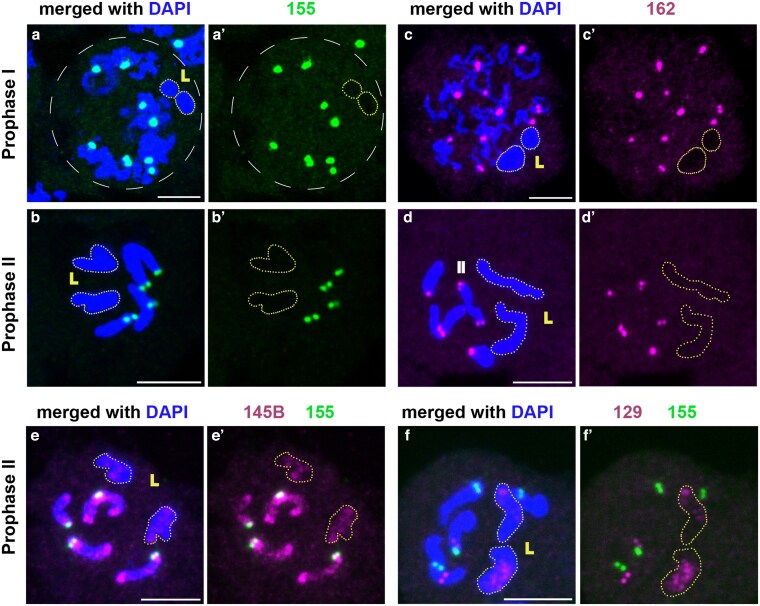
Distributions of satellite DNAs on male meiotic chromosomes. Germline-restricted (L) chromosomes are outlined. Prophase I a) and prophase II b) cells with hybridization signal from BcopSat-155 (green); prophase I c) and prophase II d) cells with hybridization signal from BcopSat-162 (magenta). e) Prophase II cells hybridized with BcopSat-155 (green) and BcopSat-145B (magenta). f) Prophase II cells hybridized with BcopSat-155 (green) and BcopSat-129 (magenta). a′–f′) show hybridization signal in the absence of DAPI. Scale bar represents 5 µm.

Because salivary gland nuclei do not have the germline-limited L chromosomes, we next examined whether the satellite DNA families found on the core chromosomes are also present on L chromosomes using DNA FISH on early pupal testes. The early pupal testis contains cells undergoing meiosis, which can be subjected to chromosome squashes and DNA FISH. L chromosomes in prophase I spermatocytes can be differentiated from X and autosomes based on their ovoid shape (Metz 1938; Rieffel and Crouse 1966) (Fig. 5, a and c). During prophase II, L chromosomes appear as large metacentric chromosomes (Fig. 5, b and d, e, f).

Although the BcopSat-155 satellite DNA was previously suggested to be present at the centromeres of all chromosomes, including L chromosomes (Escribá *et al.* 2011), we found that this satellite DNA was present only at the centromeres of the X and autosomes but not on the L chromosomes (Fig. 5, a, a′, b, and b′). The probe sequence used by Escribá *et al.* (2011) was cloned using DNA microdissection, and this sequence contained a 78 bp sequence that matches the BcopSat-155 sequence, as well as a 65 bp sequence from the *B. coprophila* genome that does not match the BcopSat-155 sequence. This 65 bp sequence appears to be unique to the DNA cloned by Escribá *et al.* (2011), but was not associated with any of BcopSat-155 sequence identified in the genome assembly, perhaps indicating this is a result of cloning artifact (2 DNA fragments joined during cloning) or that this chimeric sequence exists in the *B. coprophila* at a very low frequency. To test the possibility that this 65 bp sequence (not present in BcopSat-155 sequence) might have yielded the FISH signal on L chromosome centromeres in the earlier study (Escribá *et al.* 2011), we designed multiple probes that cover the entirety of the DNA sequence in the clone described by Escribá *et al.* (2011): the 65 bp sequence that does not match BcopSat-155 sequence, and the 78 bp sequence that matches BcopSat-155 sequence (Supplementary Fig. 4a). We found that none of probes from the 65 bp sequence hybridized to the centromeres of autosomes (Supplementary Fig. 4, b and b′, polytene chromosome DNA FISH) or L chromosomes (Supplementary Fig. 4c, DNA FISH on meiotic cells), although the signal was detected as multiple faint bands throughout the chromosomes on the polytene chromosomes (Supplementary Fig. 4, b and b′). In contrast, the probes against the 78 bp region matching the BcopSat-155 sequence (155.2, 155.3, Supplementary Fig. 4a) hybridized to the centromeres of core chromosomes, overlapping with the original 155 probe (Supplementary Fig. 4d).

These data strongly suggest that BcopSat-155 is only present on the centromeres of the core chromosomes (X and autosomes) and is absent from the L chromosomes. Although the source of the discrepancy between the present work and the previous work by Escribá *et al.* (2011) remains unclear, we note that the previous conclusion that BcopSat-155 is present on the centromeres of all chromosomes was drawn from the number of foci on chromosome spreads that did not resolve individual chromosomes (Escribá *et al.* 2011). Thus, it is possible that precociously separated sister centromeres or occasional aneuploid cells contributed to their conclusion that BcopSat-155 is present on the centromeres of core and L chromosomes. Moreover, BcopSat-162, which we found on the centromeres of X and autosomes (Fig. 2), was also not present on the L chromosomes (Fig. 5d). These results suggest that the X chromosome and autosomes may not share centromeric sequence with L chromosomes.

We used probes of the remaining satellite DNA families to the L chromosomes for DNA FISH on meiotic spreads, and found that BcopSat-145B (Fig. 5, e and e′), BcopSat-129 (Fig. 5, f and f′), and possibly also BcopSat-109 (Supplementary Fig. 5, c and c′) are present on the L chromosomes. Other satellite DNAs that are abundant on the X and autosomes (Table 1), BcopSat-176B, BcopSat-37B, and BcopSat-94, were not detected on the L chromosomes (Supplementary Fig. 5, a, a′, b, b′, d, and d′). The 382 bp repeat (BcopSat-382), which was described by Escribá *et al.* (2011), showed the expected hybridization patterns (chromosome IV and the L chromosomes) on polytene chromosomes and meiotic spreads (Supplementary Fig. 6).

### Identification of L chromosome-enriched satellite DNA

Considering that the L chromosomes are large heterochromatic chromosomes (Rieffel and Crouse 1966), it was surprising that most of the abundant satellite DNA families we identified were not detected on the L chromosomes by DNA FISH. Thus, we hypothesized that these chromosomes may contain distinct sets of satellite DNAs compared to the X and autosomes. As the genome assembly that we used to identify *B. coprophila* satellite DNA (Table 1) used male embryos and pupae from stages where most cells have already lost the L chromosomes (Urban *et al.* 2021), we speculated that L-specific satellite DNA is likely missing from this assembly. We therefore utilized the recent L chromosome assembly, which was generated by comparing somatic (heads) vs germ (testes) whole genome sequencing (Hodson *et al.* 2022). Since this assembly was generated using only short read data, we identified 10-fold fewer tandem repeats compared to the core chromosome scaffolds, which were generated from long-read data (Urban *et al.* 2021). Nonetheless, we were able to identify 3 abundant satellite DNA repeats with the unit sizes of 10, 38, and 39 bp in the L chromosome assembly (Hodson *et al.* 2022), which were at very low abundance or absent in the core chromosome scaffolds from the Urban assembly (Urban *et al.* 2021): BcopSat-10, BcopSat-38, and BcopSat-39 (Table 2).

**Table 2.** L satellite families.

Satellite family	Monomer length (bp)	Array size in Urban assembly (kb)	Amount in Urban assembly (kb)	Chromosomal location by DNA FISH	Distribution pattern on polytene chromosomes	Probe sequences
BcopSat-10	10	3	6	L and weakly on X, II, III, IV	Dispersed weak bands	10 AAAACGTATTAAAACGTATTAAAACGTATT
BcopSat-38	38	0.6–1.7	8	X, II, III, IV, L	Mix of dispersed weak and strong bands	38 AAAAATTTGGTCACTTTCGGTGTATGTTGGGCAGCGGG
BcopSat-39	39	n.f.	n.f.	X, II, III, IV, L	Mix of dispersed weak and strong bands	39 AAAATTTGGTCAC TTTCAGGGTTTGGTTGGGCAGCTGT

n.f., not found.

**Fig. 6.**
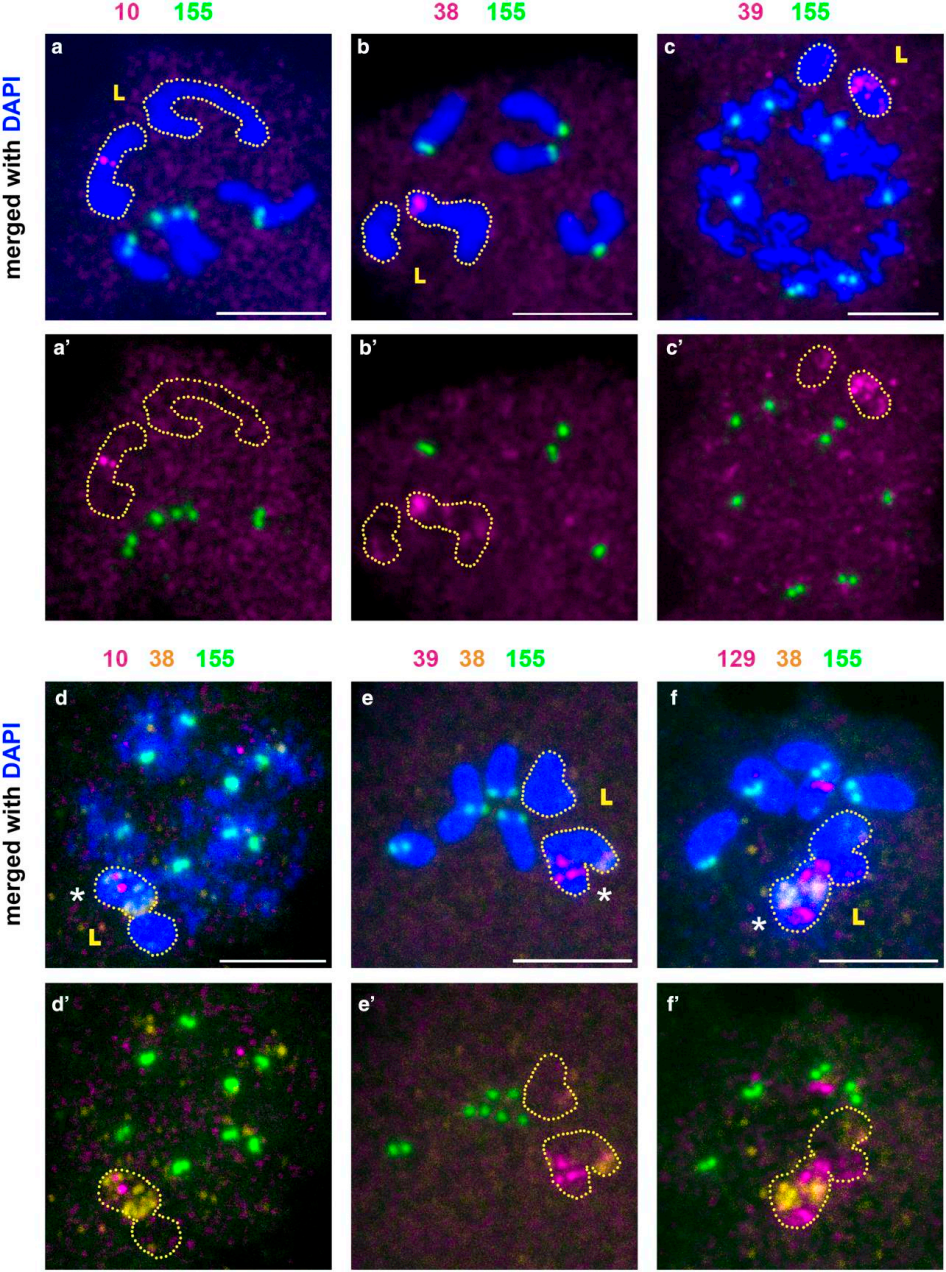
Distributions of other satellite DNAs on male meiotic chromosomes. Germline-restricted (L) chromosomes are outlined. Centromeres are shown by hybridization of BcopSat-155 (green). a) Prophase II cells with hybridization from BcopSat-10 (magenta). b) Prophase II cells with hybridization from BcopSat-38 (magenta). c) Prophase I cells with hybridization from BcopSat-39 (magenta). d) Prophase I cells with hybridization from BcopSat-10 (magenta) and BcopSat-38 (yellow). e) Prophase II cells with hybridization from BcopSat-39 (magenta) and BcopSat-38 (yellow). f) Prophase II cells with hybridization from BcopSat-129 (magenta) and BcopSat-38 (yellow). Asterisks indicate hybridization to L chromosomes. a′–f′) show hybridization in the absence of DAPI. Scale bar represents 5 µm.

We conducted DNA FISH on meiotic chromosome spreads to test whether these newly identified satellite DNAs were present on L chromosomes. We found that indeed these 3 satellite DNAs were enriched on one of the 2 L chromosomes (Fig. 6, a–c), revealing that unique subsets of satellite DNAs are present on the L vs core chromosomes. However, these probes (BcopSat-10, BcopSat-38, and BcopSat-39) detected signals at several sites on the X and autosomes of salivary gland polytene chromosomes (Supplementary Fig. 7), suggesting that these satellite DNAs are present on the X and autosomes at low abundance, which becomes only detectable with polytenized chromosomes.

### Two L chromosomes in spermatocytes are not homologs

While conducting DNA FISH on L-enriched satellite DNAs (BcopSat-10, BcopSat-38, and BcopSat-39) (Fig. 6, a–c) as well as BcopSat-129 (Fig. 5, f and f′), we noted that the abundance of these satellite DNAs noticeably differed between the 2 L chromosomes within a cell. By combining multiple probes (BcopSat-10, BcopSat-38, BcopSat-39, and BcopSat-129), we found that all of these satellite DNAs were either limited to or enriched on one of the 2 L chromosomes (Fig. 6, d–f). This result suggests that there are 2 distinct L chromosomes, rather than 2 L homologs, in *B. coprophila* male germline cells. Although previous cytological studies had assumed that the 2 L chromosomes found in germ cells were homologs (Metz 1938), a recent study (Hodson *et al.* 2022) found evidence for 2 distinct L chromosomes: First, the assembled L contigs were larger than the predicted size of a single L chromosome, and second, there was bimodality in the coverage of L contigs, implying that there were 2 separate chromosomes. Our DNA FISH experiments support this conclusion.

## Discussion

Here we utilized recent genomic data to identify previously unknown satellite DNAs in *B. coprophila*, providing a foundation for future studies on the functions of satellite DNA in this species. We have made 2 interesting observations regarding the *B. coprophila* germline-restricted L chromosomes. First, we found that the L chromosomes do not share the centromeric sequences with the core chromosomes (X and autosomes). A similar result has been found in 2 nightingale species, where a candidate centromeric satellite DNA was absent from the germline-restricted chromosome (GRC) (Ridl *et al.* 2025). It is tempting to speculate that the difference in centromeric satellite DNA may be utilized to allow for L chromosome elimination in somatic cells. For example, sequence-specific DNA binding proteins (specific for X/autosome centromeres, or L centromeres) may promote or interfere with centromere assembly in somatic cells, leading to defective kinetochore function and thus L chromosome elimination. However, cytological studies on *B. coprophila* L chromosome elimination in the soma showed that the L chromosomes appear to correctly separate at the centromeres, but the chromosome ends are not properly separated and the chromosomes are unable to segregate to the poles (Du Bois 1933; de Saint Phalle and Sullivan 1996). Such observations are apparently inconsistent with the model that loss of centromere function on the L chromosomes leads to their elimination. It is possible that chromosome elimination during embryonic divisions may involve sequences along the chromosome arms, rather than at the centromeric region. A study looking at the distribution of phosphorylated histone H3S10 during L and X chromosome elimination in early embryos found that there was strong H3S10-P signal on the arms of the chromosomes being eliminated, although H3S10-P was absent from the centromeric regions of the L and X chromosomes and the core chromosomes during elimination (Escribá and Goday 2013). These results suggest that there are differences in chromatin constitution, which may be involved in the process of chromosome elimination.

In addition to centromeric sequences, our results revealed a distinct composition of satellite DNA between the core chromosomes and the L chromosomes. It is intriguing that the core and L chromosomes do not share the centromeric sequences, raising the possibility that such distinct centromeric sequence may be involved in the L chromosome elimination. Also, this finding is consistent with the conclusion by Hodson *et al.* (2022), based on genomic evidence, that the L chromosomes did not arise from polyploidization of the X chromosomes as has been suggested previously (Haig 1993). Based on homologies found between the genes assigned to L chromosome contigs and genes from the species *Mayetiola destructor*, Hodson *et al.* (2022) proposed that the L chromosomes entered sciarids via interspecific hybridization with an ancestral member of the Cecidomyiidae family. Our findings raise the possibility that L chromosome centromeres are distinct from core chromosome centromeres because they are derived from Cecidomyiidae chromosomes. Identification of satellite DNAs in cecidomyiids in future studies will allow testing of this possibility.

Our study supports the conclusion by Hodson *et al.* (2022) that there are 2 distinct L chromosomes, which they called GRC1 and GRC2. Our cytological analysis revealed that multiple satellite DNA probes were more abundant on 1 L chromosome than the other, demonstrating that satellite DNA compositions are also distinct between 2 L chromosomes. The finding that there are 2 kinds of L chromosomes has important implications for the genetics of *Bradysia*. The egg contributes 1 L chromosome and the sperm 2 L chromosomes to the zygote, thus the embryo begins with 3 L chromosomes (Fig. 1). During early embryogenesis, all 3 chromosomes are eliminated from somatic cells, but the germ cells also eliminate one of 3 L chromosomes to have 2 L chromosomes per germ cell. Earlier studies assumed that 2 L chromosomes are homologous chromosomes; accordingly, the question “which L chromosomes are eliminated in the early germ cells?” has never been posed. The finding that there are 2 distinct L chromosomes has new implications for L chromosome elimination. As this study showed, sperm will carry both L1/GRC1 and L2/GRC2 chromosomes. Eggs carry only 1 L (L1 or L2). Then, the zygote must be L1L1L2 or L1L2L2, and the result of eliminating 1 L chromosome has 3 possible outcomes—L1L2, L1L1, or L2L2. In *B. coprophila* and many other sciarid species, females are either exclusively female-producing or male-producing, a phenomenon known as “monogenic reproduction” (Baird *et al.* 2023a). The 2 female morphs (female-producing vs male-producing) differ in the presence of a large X-linked inversion (XX′ in female-producing female, XX in male-producing female) (Crouse 1960). However, it is not currently known whether they also differ in the composition of their L chromosomes. Curiously, male-producing females and males have identical X chromosome composition in their germ cells (both are XX), hinting at the possibility that XX male germ cells and XX female germ cells may have a genetic mechanism to support spermatogenesis vs oogenesis. It is therefore tempting to speculate that L chromosome composition may be involved in sex determination in the germline, but further work is required to explore this possibility.

In conclusion, we have shown that the core chromosomes and germline-restricted L chromosomes differ in their satellite DNA composition. Notably, the L chromosomes lack the centromeric satellite DNA sequence found on all core chromosomes, and differ from the core chromosomes in the distribution and abundance of satellite DNA families. One question raised by this study is what is the nature of the centromere on L chromosomes. There are likely L-chromosome-specific satellites remaining to be discovered, and possibly one of these defines the L chromosome centromere. We were also able to distinguish 2 distinct L chromosomes based on their satellite DNA composition. We do not know whether there is functional significance to the presence of 2 different Ls; however, it is interesting that in males, the L chromosome is the only paternal chromosome that escapes elimination during meiosis I. One possibility is that the 2 different L chromosomes may be involved in germline sex determination, a question that would be addressed by determining the L chromosome composition in the female germline.

## Supplementary Material

jkaf155_Supplementary_Data

## Data Availability

A full list of tandem repeats is available in Supplementary File 1. Supplemental material available at G3 online.
